# Supplementary material for: COZOID: contact zone identifier for visual analysis of protein-protein interactions
Source: BMC Bioinformatics. 2018 Apr 6;19:125. doi: 10.1186/s12859-018-2113-6 (PMC5889581; doi:10.1186/s12859-018-2113-6)
Supplement: Supplementary file 2 — Software build. Executable binary file of the software tool. (ZIP 118,784 kb) [file 12859_2018_2113_MOESM2_ESM.zip › cozoid/java/docs/beansbinding-1.2.1-doc.zip!/org/jdesktop/beansbinding/ELProperty.html]

ELProperty (Beans Binding)


---


|  |  |  |
| --- | --- | --- |
| **Overview** \| **Package** \| **Class** \| **Use** \| **Tree** \| **Deprecated** \| **Index** \| **Help** | |  |
| **PREV CLASS**   **NEXT CLASS** | **FRAMES**    **NO FRAMES**     **All Classes** |  |
| SUMMARY: NESTED \| FIELD \| CONSTR \| METHOD | DETAIL: FIELD \| CONSTR \| METHOD |  |


---


## org.jdesktop.beansbinding Class ELProperty<S,V>

```
java.lang.Object
  org.jdesktop.beansbinding.Property<S,V>
      org.jdesktop.beansbinding.PropertyHelper<S,V>
          org.jdesktop.beansbinding.ELProperty<S,V>
```

**Type Parameters:**: `S` - the type of source object that this `ELProperty` operates on: `V` - the type of value that this `ELProperty` represents

---

``` public final class ELProperty<S,V> extends PropertyHelper<S,V> ```

An implementation of `Property` that allows Java Beans properties of
source objects to be addressed using a simple dot-separated path syntax
within an EL expression. For example, to create a simple property representing
a `Person` bean's mother's `firstName`:

```
    ELProperty.create("${mother.firstName}")
```

Note that `BeanProperty` is more suitable for
such a simple property.

To create a property representing the concatenation of a `Person` bean's
`firstName` and `lastName` properties:

```
    ELProperty.create("${firstName} ${lastName}");
```

To create a property that is `true` or `false` depending
on whether or not the `Person's` mother is older than 65:

```
    BeanProperty.create("${mother.age > 65}");
```

Paths specified in the EL expressions are resolved against the source object
with which the property is being used.

An instance of `ELProperty` is immutable and can be used with
different source objects. When a `PropertyStateListener` is added to
an `ELProperty` for a given source object, the `ELProperty`
starts listening to all objects along the paths in the expression (based on that source object)
for change notification, and reflects any changes by notifying the
listener associated with the property for that source object. So, for example,
if a `PropertyStateListener` is added to the property from the second example above
for an object `Duke`, the `PropertyStateListener` is
notified when either `Duke's` first name changes, or his last name changes.
If a listener is added to the property from the third example, the `PropertyStateListener`
is notified when either a change in `Duke's` mother or `Duke's` mother's `age`
results in a change to the result of the expression.

It is very important that any bean properties addressed via a `ELProperty`
follow the Java Beans specification, including firing property change notification;
otherwise, `ELProperty` cannot respond to change. As some beans outside
of your control may not follow the Java Beans specification, `ELProperty`
always checks the `BeanAdapterFactory` to
see if a delegate provider has been registered to provide a delegate bean to take
the place of an object for a given property. See the
ext package level documentation for more
details.

When there are no `PropertyStateListeners` installed on an `ELProperty`
for a given source, all `Property` methods act by evaluating the full expression,
thereby always providing "live" information.
On the contrary, when there are `PropertyStateListeners` installed, the beans
along the paths, and the final value, are cached, and only updated upon
notification of change from a bean. Again, this makes it very important that any
bean property that could change along the path fires property change notification.
*Note: The `setValue` method is currently excluded from the previous
assertion; with the exception of checking the cache to determine if the property is
writeable, it always evaluates the entire expression. The result of this is that
when working with paths containing beans that don't fire property change notification,
you can end up with all methods (including `getValue`) working on cached
information, but `setValue` working on the live expression. There are plans
to resolve this inconsistency in a future release.*

**Readability** of an `ELProperty` for a given source is defined as follows:
*An `ELProperty` is readable for a given source if and only if the
following is true for all paths used in the expression:
a) each bean the path, starting with the source, defines a Java Beans getter
method for the the property to be read on it AND b) each bean in the path,
starting with the source and ending with the bean on which we read the final
property, is `non-null`. The final value being `null` does not
affect the readability.*

So, in the third example given earlier, the `ELProperty` is readable for `Duke` when all
of the following are true: `Duke` defines a Java Beans getter for
`mother`, `Duke's mother` defines a Java Beans getter for
`age`, `Duke` is `non-null`, `Duke's mother`
is `non-null`. The `ELProperty` is therefore unreadable when
any of the following is true: `Duke` does not define a Java Beans
getter for `mother`, `Duke's mother` does not define a Java
Beans getter for `age`, `Duke` is `null`,
`Duke's mother` is `null`.

**Writeability** of an `ELProperty` for a given source is defined as follows:
*An `ELProperty` is writeable for a given source if and only if
a) the EL expression itself is not read-only
(ie. it is a simple expression involving one path such as "${foo.bar.baz}" AND
b) each bean in the path, starting with the source and ending with the bean on
which we set the final property, defines a Java Beans getter method for the
property to be read on it AND c) the bean on which we set the final property
defines a Java Beans setter for the property to be set on it AND d) each bean
in the path, starting with the source and ending with the bean on which we
set the final property, is `non-null`. The final value being `null`
does not affect the writeability.*

So in the first example given earlier (a simple path), the `ELProperty`
is writeable for `Duke` when all of the following are true: `Duke` defines a Java Beans getter for
`mother`, `Duke's mother` defines a Java Beans setter for
`firstName`, `Duke` is `non-null`, `Duke's mother`
is `non-null`. The `ELProperty` is therefore unreadable when
any of the following is true: `Duke` does not define a Java Beans
getter for `mother`, `Duke's mother` does not define a Java
Beans setter for `firstName`, `Duke` is `null`,
`Duke's mother` is `null`. The second and third examples above
both represent read-only ELExpressions and are therefore unwritable.

In addition to working on Java Beans properties, any object in the paths
can be an instance of `Map`. In this case, the `Map's get`
method is used with the property name as the getter, and the
`Map's put` method is used with the property name as the setter.
`ELProperty` can only respond to changes in `Maps`
if they are instances of `ObservableMap`.

Some methods in this class document that they can throw
`PropertyResolutionException` if an exception occurs while trying
to evaluate the expression. The throwing of this exception represents an abnormal
condition and if listeners are installed for the given source object,
leaves the `ELProperty` in an inconsistent state for that source object.
An `ELProperty` should not be used again for that same source object
after such an exception without first removing all listeners associated with
the `ELProperty` for that source object.

---

| **Method Summary** | |
| --- | --- |
| `static <S,V> ELProperty<S,V>` | `create(Property<S,?> baseProperty, java.lang.String expression)`             Creates an instance of `ELProperty` for the given base property and expression. |
| `static <S,V> ELProperty<S,V>` | `create(java.lang.String expression)`             Creates an instance of `ELProperty` for the given expression. |
| `V` | `getValue(S source)`             Returns the value of this `Property` for the given source. |
| `java.lang.Class<? extends V>` | `getWriteType(S source)`             Returns the type of object that is suitable for setting as the value of this `Property` by calls to `setValue`. |
| `boolean` | `isReadable(S source)`             Returns whether or not the `Property` is readable for the given source. |
| `boolean` | `isWriteable(S source)`             Returns whether or not the `Property` is writeable for the given source. |
| `protected  void` | `listeningStarted(S source)`             Called when this `PropertyHelper` changes from having no listeners installed for the given source object to having listeners installed for the given source object. |
| `protected  void` | `listeningStopped(S source)`             Called when this `PropertyHelper` changes from having listeners installed for the given source object to having no listeners installed for the given source object. |
| `void` | `setValue(S source, V value)`             Sets the value of this `Property` for the given source. |
| `java.lang.String` | `toString()`             Returns a string representation of the `ELProperty`. |

**Methods inherited from class org.jdesktop.beansbinding.PropertyHelper**

`addPropertyStateListener, firePropertyStateChange, getPropertyStateListeners, isListening, removePropertyStateListener`

**Methods inherited from class java.lang.Object**

`clone, equals, finalize, getClass, hashCode, notify, notifyAll, wait, wait, wait`

**Method Detail**

### create

```
public static final <S,V> ELProperty<S,V> create(java.lang.String expression)
```

:   Creates an instance of `ELProperty` for the given expression.

    :   **Parameters:**: `expression` - the expression **Returns:**: an instance of `ELProperty` for the given expression **Throws:**: `java.lang.IllegalArgumentException` - if the path is null or empty: `PropertyResolutionException` - if there's a problem with the expression

---


### create

```
public static final <S,V> ELProperty<S,V> create(Property<S,?> baseProperty,
                                                 java.lang.String expression)
```

:   Creates an instance of `ELProperty` for the given base property
    and expression. The expression is relative to the value of the base property.

    :   **Parameters:**: `baseProperty` - the base property: `expression` - the expression **Returns:**: an instance of `ELProperty` for the given base property and expression **Throws:**: `java.lang.IllegalArgumentException` - if the path is null or empty: `PropertyResolutionException` - if there's a problem with the expression

---


### getWriteType

```
public java.lang.Class<? extends V> getWriteType(S source)
```

:   Returns the type of object that is suitable for setting as the value
    of this `Property` by calls to `setValue`.

    See the class level documentation for the definition of writeability.

    :   **Specified by:**: `getWriteType` in class `PropertyHelper<S,V>`
    :   **Parameters:**: `source` - the source object on which to operate **Returns:**: the type of object suitable for setting as the value **Throws:**: `java.lang.UnsupportedOperationException` - if the `Property` is not writeable for the given source: `PropertyResolutionException` - if an exception occurs while evaluating the expression **See Also:**: `setValue(S, V)`, `isWriteable(S)`

---


### getValue

```
public V getValue(S source)
```

:   Returns the value of this `Property` for the given source.

    See the class level documentation for the definition of readability.

    :   **Specified by:**: `getValue` in class `PropertyHelper<S,V>`
    :   **Parameters:**: `source` - the source object on which to operate **Returns:**: the value of this `Property` for the given source **Throws:**: `java.lang.UnsupportedOperationException` - if the `Property` is not readable for the given source: `PropertyResolutionException` - if an exception occurs while evaluating the expression **See Also:**: `isReadable(S)`

---


### setValue

```
public void setValue(S source,
                     V value)
```

:   Sets the value of this `Property` for the given source.

    See the class level documentation for the definition of writeability.

    :   **Specified by:**: `setValue` in class `PropertyHelper<S,V>`
    :   **Parameters:**: `source` - the source object on which to operate: `value` - the new value for the `Property` **Throws:**: `java.lang.UnsupportedOperationException` - if the `Property` is not writeable for the given source: `PropertyResolutionException` - if an exception occurs while evaluating the expression **See Also:**: `isWriteable(S)`, `getWriteType(S)`

---


### isReadable

```
public boolean isReadable(S source)
```

:   Returns whether or not the `Property` is readable for the given source.

    See the class level documentation for the definition of readability.

    :   **Specified by:**: `isReadable` in class `PropertyHelper<S,V>`
    :   **Parameters:**: `source` - the source object on which to operate **Returns:**: whether or not the `Property` is readable for the given source. **Throws:**: `java.lang.UnsupportedOperationException`: `PropertyResolutionException` - if an exception occurs while evaluating the expression **See Also:**: `isWriteable(S)`

---


### isWriteable

```
public boolean isWriteable(S source)
```

:   Returns whether or not the `Property` is writeable for the given source.

    See the class level documentation for the definition of writeability.

    :   **Specified by:**: `isWriteable` in class `PropertyHelper<S,V>`
    :   **Parameters:**: `source` - the source object on which to operate **Returns:**: whether or not the `Property` is writeable for the given source. **Throws:**: `java.lang.UnsupportedOperationException`: `PropertyResolutionException` - if an exception occurs while evaluating the expression **See Also:**: `isReadable(S)`

---


### listeningStarted

```
protected final void listeningStarted(S source)
```

:   **Description copied from class: `PropertyHelper`**
:   Called when this `PropertyHelper` changes from having
    no listeners installed for the given source object to
    having listeners installed for the given source object. This
    is the ideal time for subclasses to install any listeners needed
    to track change on the source object.

    :   **Overrides:**: `listeningStarted` in class `PropertyHelper<S,V>`
    :   **See Also:**: `PropertyHelper.listeningStopped(S)`

---


### listeningStopped

```
protected final void listeningStopped(S source)
```

:   **Description copied from class: `PropertyHelper`**
:   Called when this `PropertyHelper` changes from having
    listeners installed for the given source object to
    having no listeners installed for the given source object. This
    is the ideal time for subclasses to remove any listeners that
    they've installed to track changes on the source object.

    :   **Overrides:**: `listeningStopped` in class `PropertyHelper<S,V>`
    :   **See Also:**: `PropertyHelper.listeningStopped(S)`

---


### toString

```
public java.lang.String toString()
```

:   Returns a string representation of the `ELProperty`. This
    method is intended to be used for debugging purposes only, and
    the content and format of the returned string may vary between
    implementations. The returned string may be empty but may not
    be `null`.

    :   **Overrides:**: `toString` in class `java.lang.Object`
    :   **Returns:**: a string representation of this `ELProperty`


---


|  |  |  |
| --- | --- | --- |
| **Overview** \| **Package** \| **Class** \| **Use** \| **Tree** \| **Deprecated** \| **Index** \| **Help** | |  |
| **PREV CLASS**   **NEXT CLASS** | **FRAMES**    **NO FRAMES**     **All Classes** |  |
| SUMMARY: NESTED \| FIELD \| CONSTR \| METHOD | DETAIL: FIELD \| CONSTR \| METHOD |  |


---
